# Super-Drained Distally Based Neurofasciocutaneous Sural Flap: A Case Series and Review of Literature

**Published:** 2015-05-12

**Authors:** Mostafa El-Diwany, Mihiran Karunanayake, Sultan Al-Mutari, Alain Duvernay, Alain Michel Danino

**Affiliations:** ^a^Department of Plastic and Reconstructive Surgery, University of Montreal Hospital Center, Montreal, Quebec, Canada; ^b^Department of Maxillofacial and Plastic Surgery, General Hospital, Teaching Hospital of Dijon, University of Burgundy, Dijon, France

**Keywords:** sural flap, neurofasciocutaneous flap, distally based flap, reconstruction, super-drainage

## Abstract

**Objective:** The distally based neurofasciocutaneous sural flap is central to the armamentarium for the reconstruction of leg's distal third, ankle, and hindfoot. Despite the use of adapted techniques aimed at increasing the flap's reliability, venous congestion remains a frequently encountered problem. We present a venous super-drainage technique used by the senior author to reduce venous congestion and improve flap reliability when harvesting larger flaps. **Methods:** A retrospective chart review, from January 2002 to October 2008, at 2 tertiary care centers, was conducted on all cases of inferior limb reconstruction with reverse sural flaps on defects greater than 10 × 5 cm. In addition, a literature review was carried out to examine the average sural flap surface area and reported complications published from 1992 to 2012. We then compared our results with those published in the literature. **Results:** A total of 15 flaps were identified. Mean flap dimensions were 14 × 8.5 cm (mean area = 115.27 cm^2^; 95% confidence interval, 99.28–131.26). None of the flaps developed complications (arterial or venous insufficiency, partial/complete necrosis). The average flap surface area in the literature is 55.08 cm^2^, with a 22% rate of total complications. We harvested significantly larger flaps (*P* < .001) with a significantly lower total complication rate (*P* < .05) when compared with that reported in the literature. **Conclusion:** Anastomosing the proximal end of the lesser saphenous vein with a vein at the defect site improves venous outflow, effectively reducing the incidence of venous congestion, increases the potential flap size, and improves reliability.

Soft-tissue defects of the hindfoot, the ankle, and the lower leg have long been viewed as challenging clinical problems due to the limited reconstruction options available for local tissue transfer. The distally based neurovascular sural flap has acquired an important role in the armamentarium for the reconstruction of the distal third of the leg, the ankle, and the hindfoot (Fig 1).

The particularity of this flap is that it is raised on the basis of the sural nerve rather than a precise vascular pedicle. The sural flap's arterial inflow is robust and constant such that arterial insufficiency is rarely encountered.^1^ However, the retrograde venous outflow is obstructed by the valves of both the lesser saphenous vein located superficially and the deep venae comitantes system; thus, venous drainage relies on avalvular communicating veins between the superficial and deep venous systems until they reach a perforator located in the distal base of the flap.^2^ Therefore, venous congestion is a frequently encountered complication, especially in large flaps, resulting in partial or total flap loss.^3^ An additional limitation of this flap has been the small size of its harvestable skin paddle, limiting its applicability.

The senior author has used a venous super-drainage technique, accomplished by anastomosing the proximal end of the lesser saphenous vein with a vein at the defect site when harvesting large sural flaps, with significant improvement in flap reliability while minimizing complications. We hypothesize that venous super-drainage reduces venous congestion and allows for the design of large flaps while ensuring reliability.

## OPERATIVE TECHNIQUE

**Figure 2:**
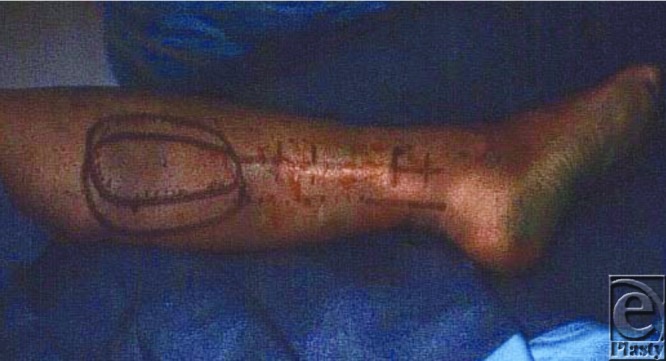
The outline of the sural flap prior to dissection.

The axis of the flap is marked preoperatively by drawing a vertical line at the pivot point located 5 cm above the lateral malleolus toward the popliteal fossa (Fig 2) while the patient is standing. The patient is placed in the prone position for surgery. The skin paddle of the sural flap is designed on the posterior calf once the defect size has been measured. The distance from the lateral malleolus to the lower aspect of the flap represents the length of the pedicle and varies according to the height of the individual and the size and location of the flap on the posterior aspect of the leg. The length of the pedicle should allow a flap transfer without tension.

The skin paddle is incised and the deep fascia is cut around the flap except at the lower border of the flap near the pedicle. An additional 3-cm incision is made at the upper border of the flap toward the popliteal fossa. Through this incision, the lesser saphenous vein is dissected superiorly and clamped to include approximately 5 cm of the saphenous vein. At the upper border of the flap, the medial sural nerve located deep into the deep fascia is also sectioned. Subfascial dissection proceeds from proximal to distal maintaining a cuff of connective tissue around the medial sural nerve and its accompanying pedicle connected to the flap's deep fascia. The pedicle is exposed along a straight line after infiltrating a 10 mL of 1% xylocaine with epinephrine in the deep dermis away from the pedicle where the skin then reflects laterally above the subcutaneous tissue.

**Figure 3:**
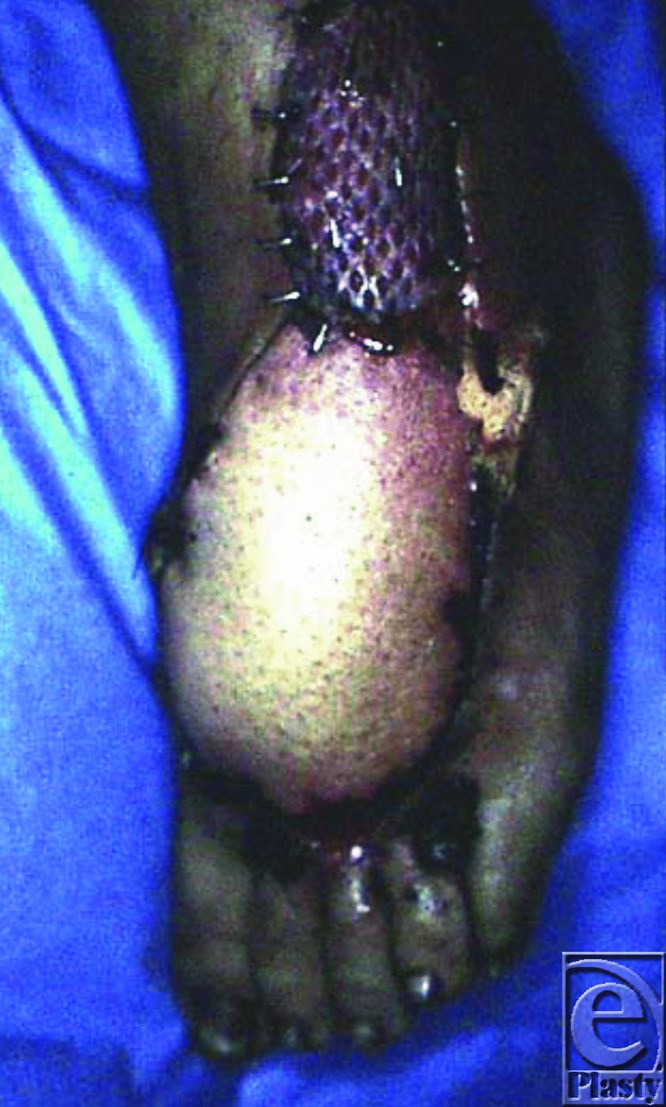
Immediate postoperative result, with split-thickness skin graft over the pedicle.

A strip of fascia with a width of 3 cm containing the sural nerve and the lesser saphenous vein is included in the pedicle. Dissection continues toward the pivot point. The flap is raised and transferred directly under a cutaneous tunnel to cover the defect. Then, an end-to-end anastomosis is performed between the free end of the lesser saphenous vein and any superficial vein with a good caliber match located in the defect site to allow for a natural anterograde venous outflow. Finally, the donor site can usually be closed primarily if the flap's width is less than 5 cm. It can also be covered by a skin graft (Fig 3).

## MATERIALS AND METHODS

From January 2002 to October 2008, a retrospective chart review, approved by our institutional review board, was conducted on patients who underwent a distally based neurofasciocutaneous sural flap reconstruction with venous super-drainage. Inclusion criteria included all patients who required a reconstruction for a defect 10 × 5 cm or greater, as they would require larger flaps and benefit the most from venous super-drainage. Informed consent was obtained prior to all procedures, and they were operated on by a single surgeon.

Demographic information including age, sex, etiology, and associated fractures was extracted. The length of the flaps, defined as the distance from the most proximal point of the skin pallet to the external malleolus, was recorded. All patients had a short hospital admission for observation of early flap-related complications. Data regarding postoperative complications, including venous congestion, infection, necrosis, and flap failure, were extracted. All patients had a minimum follow-up of 12 months.

An online literature review was carried out using Ovid MEDLINE applying the following key words: sural, distally based, reverse flap, and reconstruction. All relevant articles that used a distally based sural flap, described the flap dimensions, and provided complications were included. The mean surface area of flaps’ skin paddle was calculated and compared with our results.

Means and frequencies were calculated for all continuous and categorical variables, respectively. All continuous variables were evaluated for normality using a Shapiro-Wilk test, given the small sample sizes. Confidence intervals were calculated and a one-sample student *t* test was performed on the total surface area of the flaps. A contingency table was constructed to analyze the complication rates, and a Fisher exact test was used to compare the mean frequencies. The statistical analysis was conducted using SPSS Statistics for Windows (version 22.0.0; IBM Corp, Armonk, NY). Statistical significance was considered at *P* < .05.

## RESULTS

The study included 15 patients (7 men and 8 women), with a mean age of 42 years (range, 18–80 years). All patients were followed up after being discharged from the hospital for a mean period of 36 months. The patient demographics are listed in Table 1. The average flap dimensions were 14 cm in length (range, 10–16 cm), 8.5 cm in width (range, 5–10 cm), with an average surface area of 115.27 cm^2^ (95% confidence interval, 99.28–131.26).

Following the literature review, we retained 27 articles published on the subject between 1992 and 2012. The average area of sural flaps in the case series was 55.08 cm^2^ and was found to be significantly smaller than our average surface area (*P* < .001)^2^^,^^4^^-^29 (Table 2). The mean total rate of complications in the literature was 22%, significantly higher than our complication rate (*P* < .05). The mean flap length, as measured from the most proximal point of the skin pallet to the external malleolus, in our series was 23 cm (range, 20-24 cm).

Fourteen of our patients recovered a mobility and physical condition similar to that prior to injury. None of our patients developed partial or complete flap necrosis, or venous congestion. Only one patient, with a history of myocardial infarction, required a subsequent lower extremity amputation due to chronic peripheral vascular disease.

## DISCUSSION

The distally based neurofasciocutaneous sural flap is a valuable reconstructive option for the coverage of soft-tissue defects of varying etiologies at the level of the hindfoot, the ankle, and the lower leg. Its advantages are simplicity of design, easy and rapid dissection, short operation time, preservation of major arteries of the leg, minimal morbidity at the donor site, and the ability to complete the reconstruction in a single operation. The flap's arterial inflow is robust and constant such that arterial insufficiency is rarely encountered even in flaps with larger than average dimensions.^1^ One of the primary disadvantages of this flap is its susceptibility to venous congestion, which was demonstrated to be around 4.5% following the literature review and occurring in up to 21.4% of cases in one series.^30^ Furthermore, partial and complete flap necrosis was found to be even more prevalent with a cumulative rate of 36%.^31^ However, the case series failed to consistently report the etiology of the flap necrosis (arterial vs venous), which can further increase the actual rate of venous congestion encountered in these flaps. Large flaps have been classically associated with a greater incidence of venous congestion leading to necrosis despite adequate arterial perfusion.^32^ Other disadvantages include anesthesia over the lateral aspect of the foot associated with minimal long-term disability and an unsightly donor site scar.

It has been established that even mild congestion negatively influences flaps’ survival.^33^ Venous ischemia is in fact more damaging to tissues than arterial ischemia for an equal duration, and obstruction of venous outflow for more than 8 hours results in complete flap necrosis.^34^^,^^35^ It is the distal tip, the portion of the flap most crucial to the reconstruction, that is usually compromised first.

Various technical modifications have been proposed to prevent venous congestion including delay techniques, exteriorization of the pedicle, modification of the pivot point, etc.^31^^,^^35^^-^37 Super-drainage of the flap, a technique used by the senior author, takes advantage of the natural venous outflow course of the lesser saphenous vein and dramatically reduces or eliminates venous congestion and allows for the design of larger than average flaps while ensuring reproducible outcomes.

The average size of the flaps in our series was 14 cm in length and 8.5 cm in width, with a mean surface area of 115.27 cm^2^ compared with 55.08 cm^2^ in the literature (*P* < .001) Furthermore, in our case series, there were no complications from venous congestion or partial/complete necrosis in any of the flaps, significantly lower than that reported in the literature (*P* < .05). Therefore, the anastomosis of the lesser saphenous vein with a vein at the recipient site not only improves the flap's reliability but also allows for harvesting larger flaps. This was achieved through a reliable venous outflow with our microsurgical venous anastomosis.

**Figure 4:**
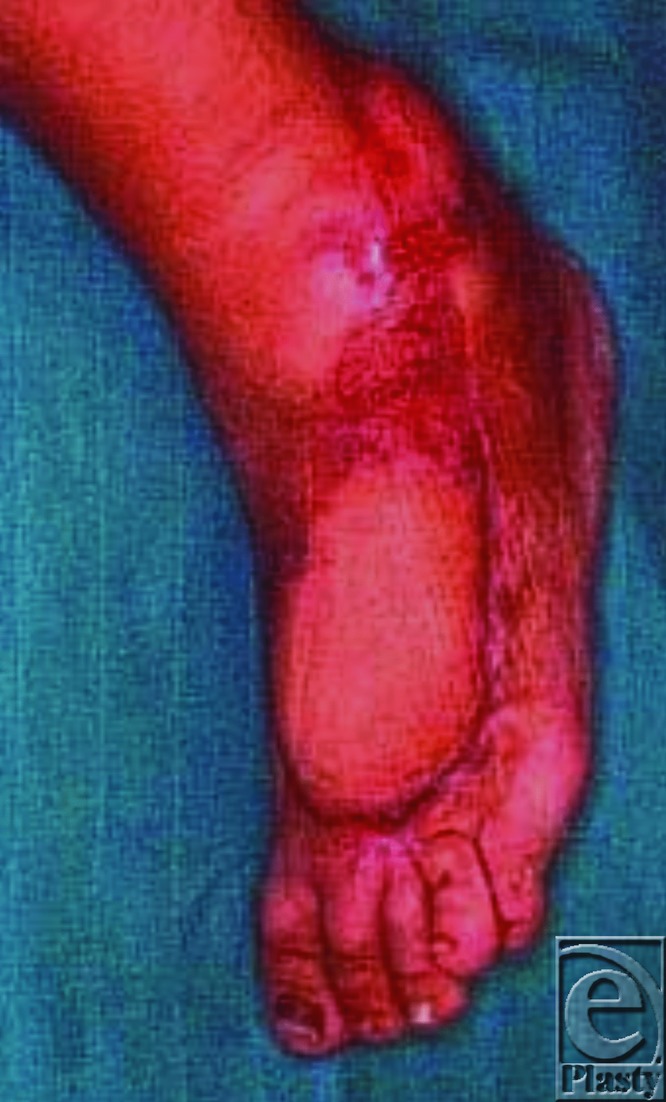
Long-term result.

Moreover, most of the previously published articles focused mainly on the flap size, omitting to mention the length of the pedicle. In our series, we were able to achieve a flap length of as long as 24 cm (mean = 23 cm), encountering neither venous congestion nor necrosis of the tip (Fig 4). We believe this result is also attributed to the concomitant improvement of the venous outflow through our super-drainage technique. We found that the pedicle's length, rather than the flap surface area, plays a fundamental role in obtaining adequate coverage by improving the maneuverability of the flap and allowing for proper inset at the defect site.

One of the primary limitations of our study is the small sample size (*N* = 15). Furthermore, given the retrospective design, there is the risk of a patient selection bias and omission of patients who had a higher risk of complications based on their comorbidities. Furthermore, we selected patients who had a minimum defect size of 10 × 5 cm, thereby omitting smaller flaps, which could have developed complications, and increasing our mean flap size. However, our goal was to evaluate the benefit of a venous super-drainage system on larger flaps, which are more prone to venous congestion and necrosis. When comparing our results with those reported in the literature, one has to keep in mind the difference in selection criteria in the other studies. Although the mean flap size calculated from the literature review was 55.08 cm^2^, this value does not necessarily reflect defects greater than 10 × 5 cm. In addition, the results of the literature review are prone to selection bias, reporting bias, and the heterogeneity of the outcomes reported, as these are low level of evidence case series. Although a prospective cohort study with a larger sample size would be ideal, it is rendered difficult by the low incidence of these defects.

## CONCLUSION

We believe that venous super-drainage could dramatically reduce the incidence of venous congestion by augmenting the venous output, thereby increasing flap reliability and allowing for larger flap sizes with lower rates of complications related to venous congestion. We advise the routine usage of this technique in flaps larger than 10 × 5 cm, especially if the flap shows signs of initial venous congestion, which puts it at an increased risk of necrosis.

## Figures and Tables

**Figure 1:**
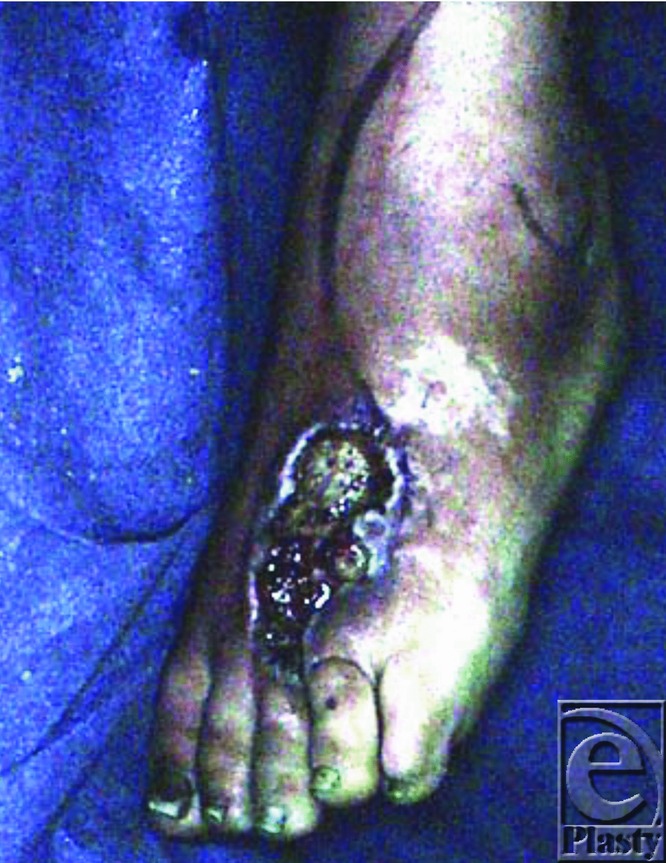
Defect site as a result of burn injury.

**Table 1:** Patients’ information and dimensions of flaps

Patient	Age	Gender	Cause of defect	Dimensions of skin pallet,^*^ cm	Flap's length,† cm	Complications
1	18	M	Fracture Gustillo IIIB	16 × 10	24	None
2	80	F	Squamous-cell carcinoma excision	10 × 8	22	None
3	45	F	Melanoma excision	16 × 8	22	None
4	55	M	Fracture Gustillo IIIB	15 × 8	22	None
5	23	M	Fracture Gustillo IIIB	15 × 10	24	None
6	24	F	Fracture Gustillo IIIB	14 × 9	23	None
7	34	M	Fracture Gustillo IIIB	15 × 9	23	None
8	55	F	Burn IIB	12 × 9	23	None
9	33	F	Burn IIB	12 × 9	23	None
10	44	F	Diabetic ulcer on the lateral aspect of the foot	11 × 9	23	None
11	45	F	Pressure ulcer on the lateral border of the foot	10 × 9	23	None
12	34	F	Contracture	10 × 5	20	None
13	43	M	Open fracture on the dorsum of the foot	15 × 10	24	None
14	42	M	Achilles tendon exposed	12 × 9	23	None
15	45	M	Infected wound after Achilles tendon reparation	13 × 9	23	None
**Summary**						
***N***	**Mean age**	**Gender**	**Etiology**	**Average dimensions (width × length)**	**Mean flap length**	**Total rate of complications**
15	41 y	0.47 M	T = 9; N = 2; C = 4	14 × 8.5 cm	23 cm	0

*Dimensions of the skin pallet (width × length).

**†**Flap's length as measured from the most proximal point of the skin pallet to the external malleolus.

M indicates male; F, female; T, traumatic; N, neoplastic; C, chronic wound.

**Table 2:** Summary of flap paddle mean surface area and complications in literature

First author	Year	No. of cases	Average flap surface area (cm^2^)	Venous congestion	Edema	Partial necrosis	Total necrosis	Other complications	Total rate of complications (%)	Notes
Masquelet^5^	1992	6	13.7	0	0	0	0	1	17	
Hasegawa^6^	1994	20	31.9	0	1	1	0	0	10	
Huisinga^7^	1998	14	48.6	1	0	2	1	0	29	
Yilmaz^8^	1998	17	56.6	2	2	2	0	0	35	
Fraccalvieri^9^	2000	18	42.3	1	0	3	0	0	22	
Al-Qattan^10^	2001	15	65.4	2	0	3	0	0	33	Inclusion of muscle “cuff”
Singh^11^	2001	7	53	0	2	3	0	0	71	
										
Ayyappan^13^	2002	5	194	1	2	1	0	0	80	Largest reported flap
Price^4^	2002	11	35.8	0	0	3	0	0	27	
Akyürek^12^	2004	2	77.5	0	0	0	0	0	0	Delay technique
Benito-Ruiz^14^	2004	11	23.8	0	0	1	1	0	18	
Karacalar^15^	2004	9	86.8	0	0	0	0	2	22	Delay technique
Chen^16^	2005	2	54.9	0	0	0	0	0	0	
Kneser^17^	2005	11	137	0	0	3	0	0	27	Delay technique
Maffi^18^	2005	7	43.2	0	0	1	0	0	14	Exteriorizing the pedicle
Rohmiller^19^	2005	11	58	0	0	3	0	0	27	
Top^20^	2005	12	55.6	1	0	0	0	0	8	Conventional and superdistally based sural flap
Tosun^21^	2005	37	47.9	0	0	4	0	5	24	Delay technique
Zhang^2^	2005	21	80.4	0	0	1	0	1	10	Modified pivot point
Buluç^22^	2006	10	30.1	0	0	3	0	0	30	
Ríos-Luna^23^	2007	14	29.5	2	0	2	0	0	29	Subcutaneous tunneling
Wong^24^	2008	14	114	0	0	1	0	0	7	Intermittent short saphenous vein phlebotomy
Ahmed^25^	2008	10	52.1	1	0	0	0	0	10	
Köse^26^	2011	10	48.2	2	0	1	0	0	30	Expanded flap
Peng^27^	2011	23	53.6	2	0	0	0	0	9	Ligation of the short saphenous vein
Hamdi^28^	2012	25	30.1	1	0	2	0	0	12	Subcutaneous tunneling
Kececi^29^	2012	11	38.5	0	0	1	0	2	27	Exteriorizing the pedicle
**Summary**										
***N***		**Total cases**	**Average flap surface area**	**Rate of venous congestion**	**Rate of edema**	**Rate of partial necrosis**	**Rate of total necrosis**	**Rate of other complications**	**Total rate of complications**	
27		353	55.08 cm^2^	4.5%	2.8%	4.2%	2.8%	3.4%	17.8%	
